# Diffusion Magnetic Resonance Imaging-Based Biomarkers for Neurodegenerative Diseases

**DOI:** 10.3390/ijms22105216

**Published:** 2021-05-14

**Authors:** Koji Kamagata, Christina Andica, Ayumi Kato, Yuya Saito, Wataru Uchida, Taku Hatano, Matthew Lukies, Takashi Ogawa, Haruka Takeshige-Amano, Toshiaki Akashi, Akifumi Hagiwara, Shohei Fujita, Shigeki Aoki

**Affiliations:** 1 Department of Radiology, Juntendo University Graduate School of Medicine, Tokyo 113-8421, Japan; christina@juntendo.ac.jp (C.A.); yuya.saito.fwd@gmail.com (Y.S.); syutucchi10@gmail.com (W.U.); t.akashi.sg@juntendo.ac.jp (T.A.); ahagiwara-tky@umin.ac.jp (A.H.); sh-fujita@juntendo.ac.jp (S.F.); saoki@juntendo.ac.jp (S.A.); 2 Department of Multidisciplinary Internal Medicine, Faculty of Medicine, Tottori University, Yonago 683-8504, Japan; katoayumy@gmail.com; 3 Department of Neurology, Juntendo University School of Medicine, Tokyo 113-8421, Japan; thatano@juntendo.ac.jp (T.H.); takashio@juntendo.ac.jp (T.O.); h-amano@juntendo.ac.jp (H.T.-A.); 4 Department of Diagnostic and Interventional Radiology, Alfred Health, Melbourne, VIC 3004, Australia; mwlukies@gmail.com

**Keywords:** Alzheimer’s disease, amyotrophic lateral sclerosis, biomarker, diffusion kurtosis imaging, diffusion tensor imaging, free-water imaging, neurite orientation dispersion and density imaging, Parkinson’s disease

## Abstract

There has been an increasing prevalence of neurodegenerative diseases with the rapid increase in aging societies worldwide. Biomarkers that can be used to detect pathological changes before the development of severe neuronal loss and consequently facilitate early intervention with disease-modifying therapeutic modalities are therefore urgently needed. Diffusion magnetic resonance imaging (MRI) is a promising tool that can be used to infer microstructural characteristics of the brain, such as microstructural integrity and complexity, as well as axonal density, order, and myelination, through the utilization of water molecules that are diffused within the tissue, with displacement at the micron scale. Diffusion tensor imaging is the most commonly used diffusion MRI technique to assess the pathophysiology of neurodegenerative diseases. However, diffusion tensor imaging has several limitations, and new technologies, including neurite orientation dispersion and density imaging, diffusion kurtosis imaging, and free-water imaging, have been recently developed as approaches to overcome these constraints. This review provides an overview of these technologies and their potential as biomarkers for the early diagnosis and disease progression of major neurodegenerative diseases.

## 1. Introduction

The prevalence of neurodegenerative diseases is increasing in parallel with the rapid increase in aging societies worldwide. For example, two of the most common neurodegenerative diseases, Alzheimer’s disease (AlzD) and Parkinson’s disease (PD), are estimated to globally impact 35 million [1] and 6 million [2] patients, respectively. Importantly, the prevalence rates of neurodegenerative diseases are projected to increase even more rapidly with the aging global population since aging is a major risk factor for these diseases, representing a growing public health crisis. There are currently no curative treatments for neurodegenerative diseases; therefore, the development of disease-modifying therapies that can prevent the progression of underlying pathological changes is eagerly anticipated. Biomarkers that can be used to detect pathological changes before the development of severe neuronal loss and consequently facilitate early intervention with disease-modifying therapeutic approaches are urgently needed. Among the numerous candidate biomarkers that have been proposed, magnetic resonance imaging (MRI) is an ideal biomarker candidate, providing a powerful approach that allows noninvasive in vivo brain evaluation. Specifically, diffusion MRI is promising because it can infer microstructural characteristics of the brain, including microstructural integrity and complexity, as well as axonal density, order, and myelination, utilizing water molecules that diffuse within the tissue with displacement at a micron scale [3]. Diffusion tensor imaging (DTI) is the most commonly used diffusion MRI technique to assess pathophysiology in neurodegenerative diseases; however, due to various limitations associated with DTI, new technologies, including neurite orientation dispersion and density imaging (NODDI), diffusion kurtosis imaging (DKI), and free-water imaging (FWI), have been recently developed as alternative approaches (see Table 1 for summary). This review provides an overview of these technologies and their potential as biomarkers for the early diagnosis and preventing the progression of typical neurodegenerative diseases.

## 2. Diffusion MRI Techniques

### 2.1. DTI

In the brain, water diffusion is affected by the biologically organized structure of the brain, which includes axons, myelin, cerebrospinal fluid (CSF), and neuronal soma and dendrites. Water diffusion in the brain can be categorized into isotropic diffusion, in which water diffuses equally in all directions (e.g., in CSF and gray matter (GM)), and anisotropic diffusion, in which water diffusion is unidirectional (e.g., in white matter (WM)). The applications and utility of DTI in diseases affecting the brain have been previously reviewed [3,4]. DTI describes brain structures based on water diffusion using four metrics: fractional anisotropy (FA), mean diffusivity (MD), axial diffusivity (AD), and radial diffusivity (RD) [5].

DTI is widely utilized in neurodegenerative diseases such as AlzD [6,7,8], PD [9,10,11,12], multiple sclerosis [13,14,15], stroke, and traumatic brain injury [16,17,18]. However, several shortcomings limit its clinical utility. First, DTI does not reflect the non-Gaussian diffusion properties of water molecules in some biological tissue components, such as the cell membrane and myelin sheath, which leads to biological restrictions [19,20]. Therefore, DTI fails to detect microstructural changes in GM, which is composed primarily of neuronal cell bodies and exhibits more isotropic water diffusion [19,20]. Second, DTI assumes that each voxel comprises a single tissue compartment, which creates a partial volume effect due to the presence of extracellular free water, such as the CSF [5], and significantly influences the accuracy of DTI measurements at the GM/WM boundary [21,22] and that of the GM voxels contaminated by the CSF [23,24]. Third, DTI parameters do not reveal disease-specific and pathological information [3]. For example, the question of whether a decrease in FA implies a reduction in the axon density or axon-bundle cross-section remains unclear and the interpretation of DTI parameters is controversial [25,26,27,28]. Finally, the DTI model is an over-simplification of the brain anatomy. Although WM voxels contain crossing fibers and comprise up to 90% of all voxels in the adult brain [29,30], DTI expresses only a single principal direction; consequently, FA decreases in such voxels even in normal brain tissue [31].

### 2.2. DKI

DKI was proposed as a mathematical extension of DTI. Kurtosis measures the degree of non-Gaussian distribution in water diffusion in a voxel and is dimensionless [19,20]. Thus, DKI detects the restriction of water diffusion due to the complexity of brain tissue components such as the cell membrane and myelin sheath [32]. The higher the diffusion kurtosis, the more the water molecule diffusion deviates from the Gaussian distribution, which indicates a more restricted diffusion environment. In contrast, lower diffusion kurtosis implies less restricted diffusion, such as that which occurs in neuronal loss [33].

DTI requires at least two b-values and six diffusion gradient directions, whereas DKI requires at least three b-values and 15 diffusion gradient directions for a more complex model [34]. DKI describes the state of brain tissue using three kurtosis metrics: mean kurtosis (MK), axial kurtosis (AK), and radial kurtosis (RK). DKI has been used to evaluate neurodegeneration in WM with complex structures, including voxels containing crossing fibers [12,35,36,37]. Moreover, DKI parameters reflect the restriction of water diffusion in anisotropic as well as isotropic environments compared to DTI parameters, which assume non-restriction of water diffusion; therefore, the utility of DKI has also been demonstrated for the evaluation of microstructural changes in GM, which is composed primarily of neuronal cell bodies and exhibits isotropic water diffusion [20,28,38,39].

Thus, DKI has several limitations despite its superiority to DTI for the evaluation of pathological changes in the brain. First, DKI’s acquisition time is longer than that of DTI (approximately 10 min) [19,40], which hinders its utility in clinical practice as the more complex DKI model requires more parameters than DTI. As another limitation, neither DKI nor DTI explains disease-specific and pathological changes such as the density, dispersion, and cross-section of axons or dendrites in neurons because neither model includes biophysical assumptions [19,20].

### 2.3. FWI

As described in Section 2.1, DTI can accurately estimate indices that reflect tissue-specific microstructures only in voxels containing a single type of brain tissue but cannot quantify tissue-specific indices in voxels contaminated by extracellular free water, such as the CSF [5,21,22,23,24]. FWI was initially proposed for the separate interpretation of brain tissue microstructures and extracellular fluid within the same voxel [41]. FWI is a two-compartment (i.e., bi-tensor) model which comprises anisotropic brain tissue and isotropic free water. FWI estimates the free water volume fraction (FW) map with the free water compartment and the conventional DTI map with the tissue compartment after removing the contamination of extracellular free water [41]. Thus, FWI can improve the accuracy of single-tensor DTI indices and specifically evaluates brain tissue microstructures after the elimination of free water. Furthermore, the free-water map is considered a promising biomarker for distinguishing between neuronal degeneration and free water accumulation in the extracellular space associated with neuronal abnormalities such as neuroinflammation [41,42,43]. FWI can quantitatively estimate the degree of edema and atrophy, as well as neuroinflammation, and therefore might provide a better understanding of the pathology underlying neurodegenerative diseases such as PD [42], schizophrenia [44,45], and depression [46].

FWI can be calculated using clinically prevalent single-shell diffusion data using the same approach as that used for DTI [41], and has demonstrated comparable accuracy with that of FWI derived from multi-shell data [47]. However, the estimation of FWI depends heavily on the regularization constraints of spatial smoothing, which may lead to lower sensitivity in subtle pathologies [41,47]. When multishell diffusion acquisition is used to estimate FWI, other algorithms that are free from spatial regularization might be able to reconstruct more specific FWI indices [47,48].

### 2.4. NODDI

NODDI was developed to provide a more specific characterization of brain tissue microstructures compared with signal representations such as DTI and DKI. NODDI models three brain tissue compartments. The intracellular compartment accounts for the space bounded by the neurite membrane, the extracellular compartment refers to the space surrounded by neurites, and the isotropic water pool reflects the space occupied by the CSF [49]. Several algorithms that assume multiple compartments, such as the composite hindered and restricted water diffusion (CHARMED) model [50,51], were developed before NODDI. However, the novelty of NODDI lies in its ability to provide the characteristics of angular variations of neurites in each voxel. NODDI not only allows the quantification of the isotropic volume fraction (ISOVF, volume fraction of extracellular isotropic free water) but also measures the orientation dispersion index (ODI, index of intracellular neurite dispersion) and the intracellular volume fraction (ICVF, neurite density) after removing the partial volume effect of extracellular free water [49,52]. Therefore, ICVF and ODI can describe the biological microstructures of axons and dendrites after the removal of extracellular free water in the voxel. Furthermore, an increased ISOVF in WM might also explain neurodegeneration accompanied by an increase in extracellular isotropic fluid, such as in the case of neuroinflammation [49].

Accordingly, NODDI may be used to represent microstructures more specifically compared with the signal representation techniques [49,53,54], although NODDI has several limitations. First, although ODI is highly accurate in estimating single-shell diffusion data, ICVF and ISOVF require multi-shell diffusion data, with a minimum of two shells, similarly to DKI [49,55]. Therefore, a long acquisition time is necessary in order to estimate neurite density and extracellular fluid. Second, although the anisotropic orientation dispersion of neurites caused by bending and fanning fibers is shown in the entire brain [56], NODDI cannot evaluate the complex neurite anisotropic dispersion because it models only the isotropic dispersion of neurites.

## 3. Applications of Advanced Diffusion MRI Techniques in Neurodegenerative Diseases

### 3.1. AlzD

AlzD is the most common cause of dementia, accounting for 50–60% of all cases, and is characterized by the widespread accumulation of neuritic plaques composed of amyloid-β (Aβ) and neurofibrillary tangles comprising hyperphosphorylated tau [57]. The apolipoprotein E (APOE) ε4 allele has also been reported to account for most of the genetic risk in sporadic AlzD [58]. Subjects with a risk gene for AlzD are considered to have preclinical AlzD [59], whereas those with mild cognitive impairment (MCI) are considered to have early-stage AlzD [60]. Atrophy of the medial temporal lobe is regarded as a classical imaging finding in AlzD; however, this MRI structural biomarker is not very sensitive in the early stages of AlzD [61]. Numerous studies have reported the advantages of diffusion-derived microstructural metrics over morphological volumetry in AlzD.

#### 3.1.1. DTI in AlzD

A large-cohort multicenter study including DTI data collected from nine different scanners showed significantly decreased FA and increased MD in areas with AlzD pathology, including the corpus callosum, cingulate gyrus, fornix, precuneus, medial and lateral temporal lobes, and prefrontal lobe WM [8]. However, structural medial temporal lobe volumetry remains superior to DTI in the detection of early-stage AlzD [6].

#### 3.1.2. DKI in AlzD

Yuan et al. [62] performed whole-brain voxel-based analyses in patients with early AlzD. Compared to the controls, the patients with AlzD exhibited decreased MK, AK, and RK in their WM, including the genu of the corpus callosum, bilateral cingulum bundles, and bilateral temporal and frontal regions, as well as in GM, including the bilateral temporal cortices, parahippocampal gyrus, hippocampus, cingulate gyrus, thalamus, and amygdala. Although the changes in DKI and DTI metrics largely overlapped, only MK could detect significant differences in the lentiform nucleus between the patients with AlzD and the controls. The lentiform nucleus of the striatum is one of the neuronal circuits affected during disease progression in AlzD. Furthermore, in the patients with AlzD, the MK values of the genu of the corpus callosum, left cingulate bundle, and bilateral temporal and frontal lobes were significantly and positively correlated with mini-mental state examination (MMSE) scores. Similarly, Gong et al. [63] observed a positive correlation between the MK of WM of occipital lobe and MMSE score. In addition, a negative correlation between AK and MMSE scores was also observed in the WM of the parietal and occipital lobes and in the GM of the occipital lobe.

Utilizing both region-of-interest (ROI) analyses and tract-based spatial statistics (TBSS), Dong et al. [64] evaluated patients with AlzD categorized into those with low, intermediate, and high Aβ levels, defined by mean standardized uptake values of ≤1.00, 1.00–1.17, and ≥1.17, respectively, based on positron emission tomography (PET)-MRI with ^18^F-florbetapir.

**Figure 1:**
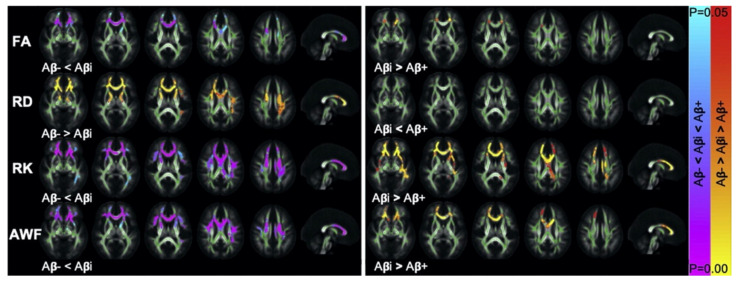
Tract-based spatial statistics. Axial and midsagittal views show significant differences in FA (**row 1**), RD (**row 2**), RK (**row 3**), and AWF (**row 4**) between patients with low Aβ (Aβ^−^)/intermediate Aβ (Aβi) and Aβi/high Aβ (Aβ^+^) levels. Clusters of increases (red/orange) and decreases (blue/purple) in Aβ levels are overlaid on the FA template, together with the mean skeleton (green). The observed differences between the Aβ^−^ and Aβi groups are in the opposite direction to those observed between the Aβi and Aβ^+^ groups. Tract-based spatial statistics reveals significant differences involving the genu of the corpus callosum and the anterior corona radiata. Directions of changes are consistent with those observed in region-of-interest analysis. Abbreviations: Aβ, amyloid-β; AWF, axonal water fraction; FA, fractional anisotropy; RD, radial diffusivity; RK, radial kurtosis (adapted and reproduced with permission by Dong et al. [64]).

Interestingly, the authors found a greater degree of diffusion restriction, based on higher RK, in the intermediate Aβ group compared to the low Aβ group (Figure 1). The authors also reported a lower degree of diffusion restriction, reflected by lower RK, mainly in the genu of the corpus callosum and fornix in the intermediate Aβ group compared to the high Aβ group. The nonmonotonic trend of diffusion changes observed with increasing amyloid burden suggests that inflammation is an early event in AlzD and that neurodegeneration worsens with disease progression.

Wang et al. [65] evaluated between-group differences in DKI and DTI metrics of the subcortical nuclei between patients with mild and moderate/severe AlzD. Increased MD values were observed in the left hippocampus, left amygdala, and right caudate in the mild AlzD group and in the bilateral hippocampi and right amygdala in the moderate/severe AlzD group. However, decreased MK values were observed only in the bilateral hippocampi in the moderate/severe AlzD group. In contrast, Gong et al. [66] observed lower MK in the bilateral hippocampi, thalamus, putamen, and globus pallidus in patients with MCI relative to controls, whereas increased MD was detected only in the amygdala. The discrepancy between the two studies might be due to variations in acquisition parameters, sample size, and demographic and clinical data, which warrant further investigation. Nevertheless, both studies [65,66] suggested that in the early stage of AlzD, diffusion metrics exhibited significant abnormalities in more nuclei than what was detected with volumetric analysis. In later stages of AlzD, more nuclei exhibited substantial decreases in volume, whereas fewer nuclei exhibited significant differences in diffusion metrics compared to the changes observed in the early disease stage. The microstructural alterations in subcortical nuclei that were detected by means of diffusion metrics probably predated volumetric alterations observed at the macroscopic level. Interestingly, MD was shown to exhibit a higher sensitivity in capturing cortical abnormalities that primarily involve cell bodies such as astrocytes, especially in the posterior cingulate in patients with MCI and AlzD [66].

Cognitive impairment in AlzD is hypothesized to arise from brain regions that myelinate later in development [67]. In their study using WM tract integrity metrics derived from DKI to evaluate patients with amnestic MCI or AlzD and controls, Benitez et al. [68] found decreased axonal and myelin integrity in late-myelinating WM tracts of patients with AlzD compared to those with amnestic MCI and controls. In contrast, no changes were observed in early-myelinating WM tracts. Furthermore, the authors reported a correlation between the WM tract integrity metrics in late-myelinating WM tracts and semantic verbal fluency in the AlzD group.

**Figure 2:**
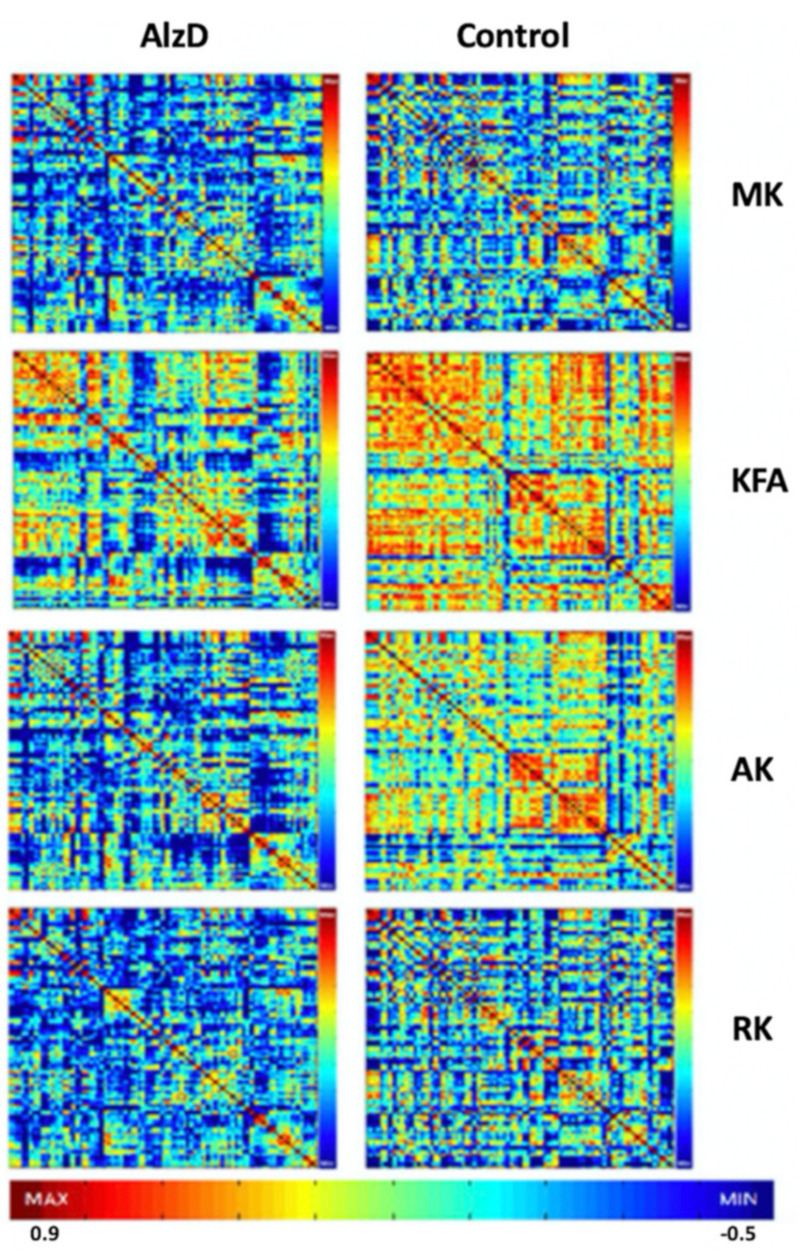
Interregional correlation matrix (90 × 90) for the DKI metrics of MK, KFA, AK, and RK in patients with AlzD and controls. Color bar represents the scale of correlations for interregional parameters. Red color represents higher positive correlation values and blue color represents higher negative correlation values. The maps reveal a great degree of dispersion in DKI observed in patients with AlzD. Note that stronger positive coordinated effects are present in extensive brain regions, indicated by red color for the metrics of MK, KFA, AK, and RK, in the control group compared with the AlzD group, and KFA is typical. A higher KFA value indicates a more compact histological structure. Abbreviations: AK, axial kurtosis; AlzD, Alzheimer’s disease; DKI, diffusion kurtosis imaging; KFA, kurtosis fractional anisotropy; MK, mean kurtosis; RK, radial kurtosis (adapted and reproduced with permission from Cheng et al. [69]).

Using graph theoretical approaches, Cheng et al. [69] investigated whole-brain network properties of the DKI metrics in AlzD and demonstrated decreased characteristic path length and increased global efficiency, based on calculations using MK, AK, and RK, in patients with AlzD compared to normal controls (Figure 2). Specifically, the GM network contained N nodes and K edges including 90 cortical and subcortical regions (45 for each hemisphere) parcellated using the Automated Anatomical Labeling atlas. The authors also suggested that the DKI networks were more closely associated with cognitive impairment than the DTI networks.

The clinical manifestations of subcortical ischemic vascular disease substantially overlap with AlzD. Tu et al. [70] showed significantly lower RK and MK in the whole thalamus, as well as in segregated thalamic regions of patients with subcortical ischemic vascular disease compared to those with AlzD. The authors also demonstrated that the DKI metrics were less affected by WM hyperintensities and were more sensitive than the DTI metrics. Using a support vector machine-based approach, Chen et al. [71] also indicated that DKI displayed a significantly better performance than DTI in the detection of AlzD-related pathological changes in WM, particularly in the hippocampus and cingulum. However, the authors suggested that the combination of DKI and DTI would increase accuracy up to 96.23%. Falangola et al. [72] utilized receiver-operating characteristic (ROC) and binary logistic regression analyses and reported that MK and RK in anterior corona radiata were the best individual discriminators between patients with MCI and normal controls and that AD in the hippocampus was the best discriminator between patients with MCI and those with AlzD.

#### 3.1.3. FWI in AlzD

In a multi-cohort study, Ofori et al. [73] demonstrated that early MCI patients had increased FW in the hippocampus compared to controls, although there was no difference in hippocampal volume between the groups. Furthermore, increased FW in the hippocampus was associated with low CSF Aβ_1–42_ levels and high global Aβ PET values.

In another study on WM, increased FW was present in the corticospinal tract, cingulum, and fornix in patients with MCI and AlzD [74] and in the transcallosal WM tracts in patients with AlzD [75] compared to controls. In another study, increased FW in the fornix was associated with memory impairment in early MCI [76]. Notably, a significant positive correlation was demonstrated between FW and AlzD-related CSF biomarkers, particularly in the temporal lobe [59]. A widespread increase in RD [77] was also observed in the WM of patients with MCI who diagnostically converted to AlzD compared to those with stable MCI [77]. The changes in FW in patients with AlzD were maintained even after WM hyperintensities were removed, indicating that FW was not associated with WM hyperintensities [75]. In line with these results, Ji et al. [78] also showed increased FW in normal-appearing WM, but not in WM hyperintensities, in patients with AlzD harboring cerebrovascular disease compared to those without cerebrovascular disease.

**Figure 3:**
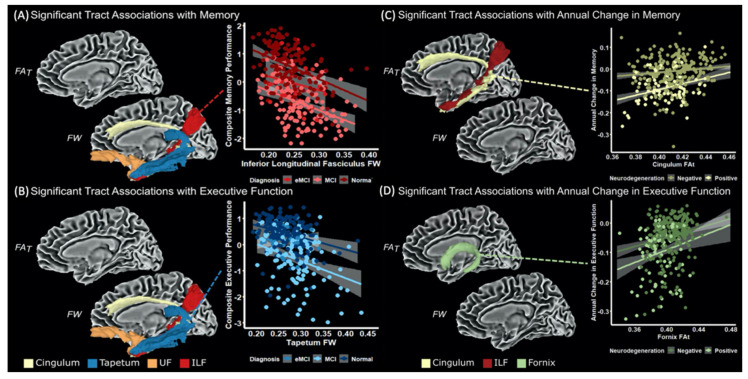
Baseline tract associations with composite memory and executive function (**A**,**B**). The medial temporal lobe tract measures exhibiting associations with memory (**A**) and executive function (**B**) include FW in the cingulum bundle, tapetum, UF, and ILF. The association of ILF FW with memory performance (**A**) and the association of tapetum FW with executive function performance (**B**) are shown. Baseline tract hippocampal interaction on annual change in memory and executive function (**C**,**D**). The medial temporal lobe tract measures exhibiting significant interaction with hippocampal volume for annual change in memory include FA_T_ in the ILF and cingulum bundle (**C**). For annual change in executive function performance, temporal tract measures exhibiting significant interaction with hippocampal volume include FA_T_ in the fornix (**D**). Groups with and without hippocampal neurodegeneration groups (positive and negative groups, respectively) are based on the previously identified cut-off volume (positive: volume ≤6723 mm^3^ [81]). Abbreviations: eMCI, early mild cognitive impairment; FA_T_, free water-corrected FA; FW, free water; ILF, inferior longitudinal fasciculus; MCI, mild cognitive impairment; UF, uncinate fasciculus (adapted and reproduced with permission from Archer et al. [79]).

Archer et al. [79] evaluated the baseline and longitudinal trajectory data on FW and free water-corrected FA (FA_T_) within medial temporal WM tracts, including the cingulum bundle, fornix, tapetum, inferior longitudinal fasciculus, and uncinate fasciculus. At baseline, FA_T_ in the tapetum and fornix and FW in all tracts were associated with hippocampal volume. Additionally, FW was negatively correlated with memory and executive function in the inferior longitudinal fasciculus, tapetum, uncinate fasciculus, and cingulum. Furthermore, there were significant interactions between hippocampal volume and medial temporal lobe WM tract FA_T_ on longitudinal cognitive trajectories (memory—inferior longitudinal fasciculus and cingulum, executive function—fornix), whereby individuals with lower hippocampal volume and WM tract FA_T_ exhibited greater longitudinal decline (Figure 3). Finally, Aβ-associated longitudinal increases in FW in the right uncinate fasciculus and the inferior longitudinal fasciculus and Aβ-associated longitudinal decreases in FA_T_ in the right uncinate fasciculus were also reported in cognitively normal elderly individuals with high Aβ burdens [80].

**Figure 4:**
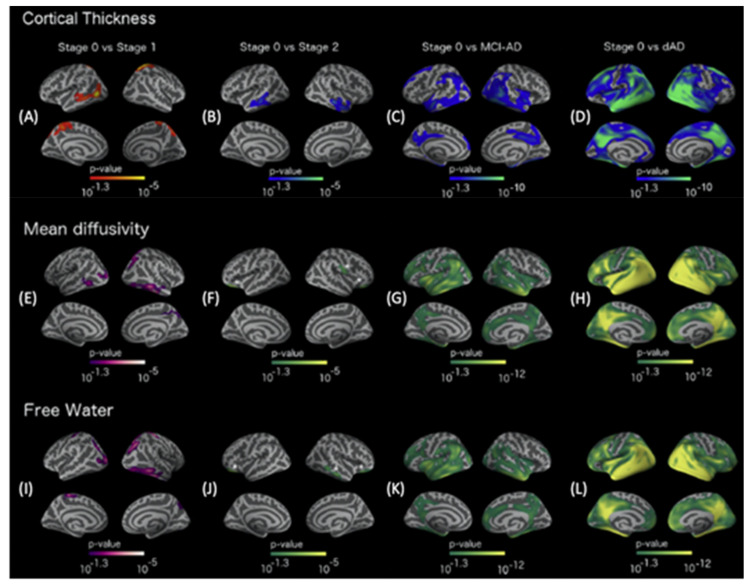
Cortical thickness patterns in the AlzD continuum. Differences in cortical thickness between stage 0 and stage 1 control subjects (**A**), stage 0 and stage 2 control subjects (**B**), stage 0 control subjects and patients with MCI-AD (**C**), and stage 0 control subjects and patients with dAD (**D**). Only clusters that survived familywise error correction (*p* < 0.05) are shown. All analyses are adjusted for age, sex, center, and APOE ε4 status. Cortical MD patterns in the AlzD continuum. Differences in MD between stage 0 and stage 1 control subjects (**E**), stage 0 and stage 2 control subjects (**F**), stage 0 control subjects and patients with MCI-AD (**G**), and stage 0 control subjects and patients with dAD (**H**). Only clusters that sur-vived familywise error correction (*p* < 0.05) are shown. All analyses are adjusted for age, sex, and APOE ε4 status. FW patterns in the AlzD continuum. Differences in FW between stage 0 and stage 1 control subjects (**I**), stage 0 and stage 2 control subjects (**J**), stage 0 and MCI-AD patients (**K**), and stage 0 control subjects and patients with dAD (**L**). Only clusters that survived familywise error correction (*p* < 0.05) are shown. All analyses are adjusted for age, sex, and APOE ε4 status. For visualization, different color codes are used for the MD, FW, and cortical thickness patterns. For MD and FW, a green-yellow color code is used; purple and white colors represent positive and negative significant values, respectively. For cortical thickness, a blue gradient scale is used as a color code; red and yellow colors represent negative and positive significant values, respectively. In the comparison between stage 2 and 0 control subjects, significant clusters are high-lighted with an asterisk to facilitate visualization. Abbreviations: AD, Alzheimer’s disease; APOE, apolipoprotein E; dAD, Alzheimer’s disease dementia with evidence of an underlying Alzheimer’s disease-related pathophysiological process; FW, free-water volume fraction; MCI-AD, mild cognitive impairment with evidence of an underlying Alzheimer’s dis-ease-related pathophysiological process; MD, mean diffusivity (adapted and reproduced with permission from Montal et al. [82]).

Cortical thickness, MD, and FW follow biphasic changes in trajectory in AlzD. In early preclinical AlzD (stage 1), cortical thickening and decreased MD and FW are correlated with amyloid deposition (Figure 4). In contrast, in late preclinical (stage 2/3) and symptomatic AlzD, increases in cortical MD, FW, and atrophy are observed in areas typically affected by AlzD [82].

#### 3.1.4. NODDI in AlzD

**Figure 5:**
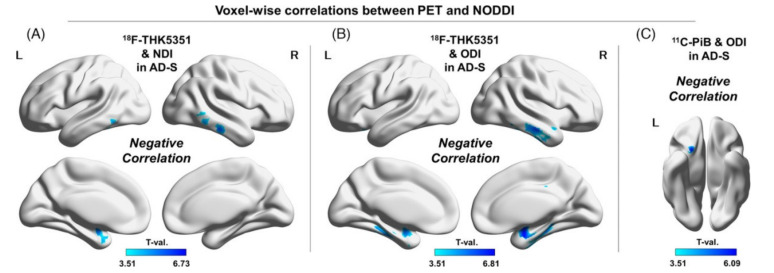
Voxel-wise correlations between PET and NODDI. Note the negative correlation between ^18^F-THK5351 accumulation and NDI in the AD-S group (**A**), the negative correlation between ^18^F-THK5351 accumulation and ODI in the AD-S group (**B**), and the negative correlation between ^11^C-PiB accumulation and ODI in the AD-S group (**C**). Color bars denote statistically significant T-values ranging from minimum to maximum. Abbreviations: AD-S, Alzheimer’s disease spectrum; NDI, neurite density index; NODDI, neurite orientation dispersion and density imaging; ODI, orientation dispersion index; PET, positron emission tomography (adapted and reproduced with permission from Sone et al. [83]).

**Figure 6:**
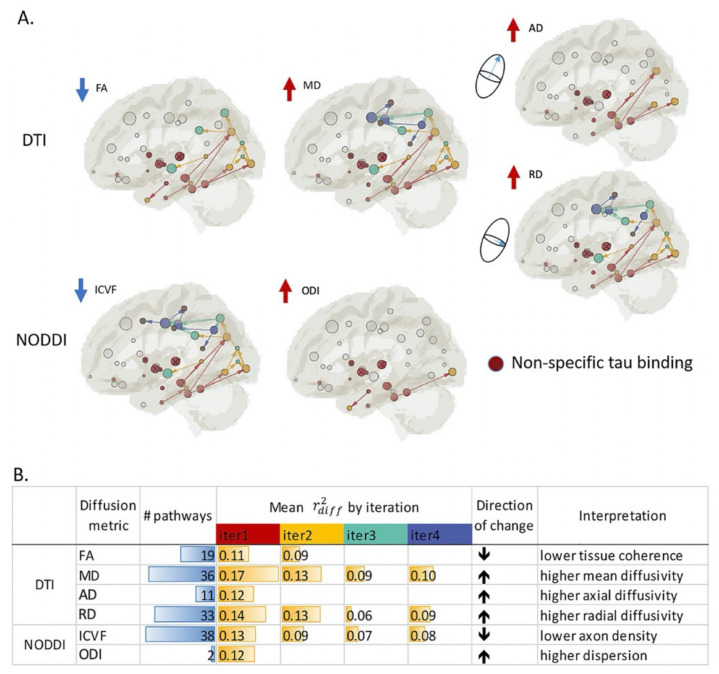
Summary of identified patterns (**A**) and statistics (**B**) for all diffusion metrics. (**A**) Among the DTI-derived metrics, higher tau-PET signals are consistently associated with decreased FA and increased MD, AD, and RD. Among the NODDI-derived metrics, higher tau-PET signals are associated with decreased ICVF, a proxy for axonal density. Increased ODI is detected in only two pathways. Independent data-driven iterative searching on AD and ICVF reveals patters similar to that observed with MD. (**B**) Statistics of the association patterns. “# pathways” denotes the total number of detected pathways that contain a dual association between tau on both ends and a diffusion metric in the connection (i.e., color-coded connections in (**A**). Adjusted r^2^_diff_ describes the additional variance in tau-PET signal explained by a diffusion metric in a multivariate regression model that controls for age and sex in the detected pathways. Only significant r^2^_diff_ values with a *p*-value of <0.05 are listed. “Direction of change” denotes the direction of change in the diffusion metric in the setting of increased tau-PET signal in adjacent regions of interest in the gray matter. Abbreviations; AD, axial diffusivity; DTI, diffusion tensor imaging; FA, fractional anisotropy; ICVF, intracellular volume fraction; iter, iteration; MD, mean diffusivity; NODDI, neurite orientation dispersion and density imaging; ODI, orientation dispersion index; PET, positron emission tomography; RD, radial diffusivity (adapted and reproduced with permission from Wen et al. [86]).

Recently, Sone et al., investigated the association between PET metrics using the novel tracer ^18^F-THK5351 and NODDI metrics in AlzD-spectrum participants and controls [83]. ^18^F-THK5351 was employed as a tracer of tau pathology and astrogliosis-related neuroinflammation [84,85]. In the amyloid-positive participants, the authors observed significant negative correlations between ICVF and ^18^F-THK5351 and between ODI and ^18^F-THK5351, mainly in the bilateral mesial and lateral temporal lobes (Figure 5), suggesting that tau and neuroinflammation in AlzD might reduce neurite density and orientation dispersion. Wen et al. [86] also found a high degree of association between the GM tau-PET signal and WM integrity measured by DTI and NODDI metrics (Figure 6). In line with Braak staging in AlzD, the spatial association pattern was initiated from the medial temporal lobe and developed posteriorly to the occipital brain regions and superiorly and anteriorly to the parietal and frontal lobes. Furthermore, the association of CSF Aβ with decreased ICVF and increased ISOVF was reported by Reas et al. [87].

Using TBSS, Fu et al. [88] found significantly decreased ICVF and ODI and significantly increased ISOVF in patients with MCI and AlzD, with broader alterations observed in those with AlzD. In the absence of a significant difference in FA between the MCI and AlzD groups, the authors suggested the superiority of NODDI metrics over DTI metrics. In contrast, in a study with a larger sample size, Wen et al. [89] showed that the DTI metrics were more sensitive compared to the NODDI metrics in detecting WM alterations in patients with MCI. Nevertheless, higher ODI values in the cingulum, thalamic radiation, and forceps major may provide complementary information on the underlying pathology in MCI. Utilizing ROI analyses and GM-based spatial statistics (GBSS), Vogt et al. [90] demonstrated lower ICVF in patients with MCI throughout temporal and parietal cortices, whereas no significant difference was found in cortical thickness. In patients with AlzD, lower ICVF and ODI were observed in more areas throughout the parietal, temporal, and frontal cortices.

Early-onset AlzD (defined as age of symptom onset <65 years) often poses a diagnostic challenge and is more likely to present with nonamnestic phenotypes [91]. Compared to controls, patients with early-onset AlzD exhibit lower ICVF in cortical areas associated with early atrophy in AlzD, such as the entorhinal cortex, inferior and middle temporal gyri, fusiform gyrus, and precuneus, whereas lower ODI is evident in the inferior and middle temporal gyri, fusiform gyrus, and precuneus [92]. Furthermore, cortical ICVF in patients with early-onset AlzD group is positively correlated with MMSE score.

Mole et al. assessed the WM [93] and GM of apolipoprotein E (APOE) ε4 carriers and noncarriers using NODDI. The authors reported lower ICVF in the right parahippocampal WM of the APOE ε4 carriers with obesity compared to the controls; however, no significant differences were observed in the cortical-subcortical GM regions between the APOE ε4 carriers and noncarriers. Similarly, Evans et al. [94] found no changes in the NODDI metrics of hippocampal GM between the APOE ε4 carriers and noncarriers. Furthermore, vascular risk factors are recognized for their crucial role in the etiology of AlzD [95]. Using NODDI, Badji et al. [96] demonstrated the association of ICVF and ISOVF with arterial stiffness, as measured by carotid-femoral pulse wave velocity, in the body of the corpus callosum. A decrease in neuronal density in the body of the corpus callosum (indexed by ICVF) was also associated with a decline in cognitive flexibility. These results suggest that arterial stiffness may cause abnormalities in WM integrity that impact cognition in elderly individuals. Midlife obesity has also been considered as a risk factor of AlzD. However, Metzler-Baddeley et al. [97] indicated that visceral fat-related systemic inflammation might damage myelin integrity, but not neurite microstructure, in limbic systems, indicated by the lack of significant changes in NODDI metrics.

### 3.2. PD

PD is the second most common neurodegenerative disease. The main motor features of PD, including bradykinesia, rigidity, and tremor, are caused by the selective loss of dopaminergic neurons in the substantia nigra (SN) pars compacta and the broad aggregation of α-synuclein-immunoreactive inclusions as Lewy neurites and Lewy bodies [98,99]. Beyond dopamine-related impairment, PD also involves the noradrenergic, serotonergic, and cholinergic neurotransmitter pathways, which are implicated in a large number of non-motor symptoms [100].

#### 3.2.1. DTI in PD

Recent meta-analyses indicate that increased MD and/or decreased FA in the corpus callosum, SN, frontal lobe, and cingulate and temporal cortices were able to differentiate patients with PD from controls [9,10]. However, DTI metrics are not considered to be diagnostic biomarkers in the early stage of PD [11,101].

#### 3.2.2. DKI in PD

**Figure 7:**
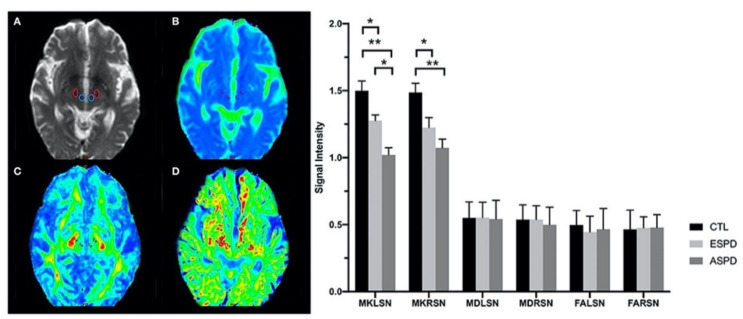
Left panel: Volumes of interest in bilateral red nuclei and the substantia nigra (**A**). Data were obtained from maps of mean diffusivity (**B**), fractional anisotropy (**C**), and mean kurtosis (**D**). Right panel: Diffusion kurtosis imaging values in the substantia nigra for CTL, ESPD, and ASPD groups. ** *p* < 0.01, ASPD vs. CTL; * *p* < 0.05, ESPD vs. CTL, ASPD vs. CTL, and ASPD vs. ESPD. Abbreviations: ASPD, advanced-stage Parkinson’s disease; CTL, control; ESPD, early-stage Parkinson’s disease; FALSN, fractional anisotropy of left substantia nigra; FARSN, fractional anisotropy of right substantia nigra; MDLSN, mean diffusivity of left substantia nigra; MDRSN, mean diffusivity of right substantia nigra; MKLSN, mean kurtosis of left substantia nigra; MKRSN, mean kurtosis of right substantia nigra (adapted and reproduced with permission from Guan et al. [102]).

Guan et al. [102] showed decreased MK in bilateral SN in patients with early-stage (Unified PD Rating Scale [UPDRS]-III score <30) and advanced-stage (UPDRS-III score ≥30) PD compared to controls (Figure 7). Moreover, patients with advanced-stage PD showed lower MK in the left SN compared to those with early-stage PD and lower MK in bilateral red nuclei compared to controls. In patients with PD, MK in the globus pallidus and red nucleus; AK in the SN, globus pallidus, and red nucleus; and RK in the globus pallidus were positively correlated with MMSE score [103]. Furthermore, reduction in the FA of kurtosis over two years was correlated with a daily L-dopa equivalent dose in the putamen of patients with PD [104]. It is worth noting that the combined used of DKI and quantitative susceptibility mapping techniques showed a sensitivity of 83–100% and a specificity of 81–100% for discriminating PD from Parkinsonisms [105,106].

Kamagata et al., evaluated changes in DKI metrics in the WM of patients with PD using tract-specific [107] and TBSS [12] analyses. Decreases in FA and MK were observed in the anterior cingulum of patients with PD, in whom MK exhibited better diagnostic performance [107]. Given that the early pathological changes in PD are present in the anterior cingulum, these findings support the utilization of DKI metrics as early PD biomarkers. Using TBSS, a reduction in MK was found in broader WM areas, including the corpus callosum and the frontal, parietal, and occipital lobes, compared to the reduction in FA observed in patients with PD [12]. Furthermore, decreased MK was also shown in WM areas with crossing fibers, such as the superior longitudinal fasciculus and corona radiata, whereas similar changes in FA were not observed in these areas.

Ischemic lesions of the striatum may affect the SN and accelerate the progression of PD [108]. Indeed, Zhang et al. [109] showed significantly higher MK in the SN of patients with PD and striatal silent lacunar infarction (SSLI) compared to those without SSLI. In a subsequent study, Zhang et al. [110] also indicated that hyperhomocysteinemia, which is frequently found in patients with SSLI, was associated with microstructural changes in the SN of patients with PD. The authors also found that the variations in changes in several outcomes between the baseline and the two-year follow-up, including increases in MK of the SN, Hoehn and Yahr staging, and UPDRS-III score, were significantly higher in patients with hyperhomocysteinemia than in those without hyperhomocysteinemia within the cohort of patients with PD.

#### 3.2.3. FWI in PD

Accumulating evidence indicates FW as a promising imaging biomarker to distinguish PD patients from normal controls. Previous studies with single- and multi-site cohorts have consistently demonstrated increased FW in the posterior SN of patients with PD compared to controls [111,112,113,114]. Longitudinal studies also showed increases in FW in the posterior SN over time, for up to four years, with no changes observed in controls [111,115,116]. A negative correlation was also demonstrated between FW in the posterior SN and ^11^C-dihydrotetrabenazine binding in the putamen, which was associated with higher Movement Disorder Society-UPDRS-III scores, Hoehn and Yahr stage, and posture and gait subscores [117].

**Figure 8:**
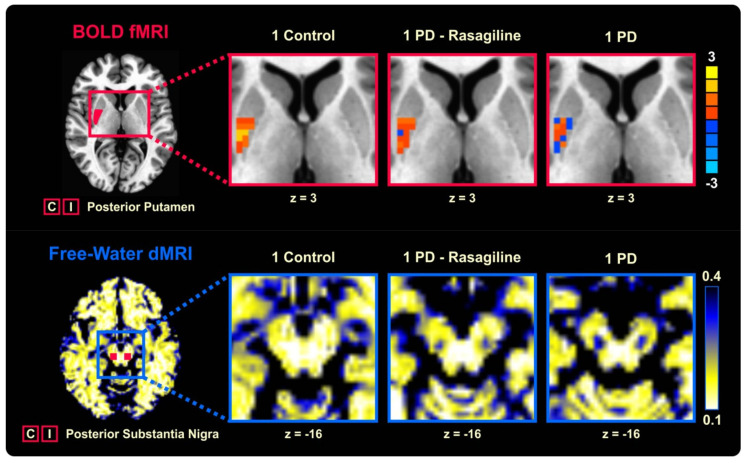
Task-based fMRI signal in the posterior putamen contralateral to the tested hand and free water volume fraction in the posterior substantia nigra averaged across sides, plotted for one control subject, one patient with PD treated with rasagiline, and one patient with PD not treated with rasagiline. Abbreviations: BOLD, blood oxygen-level dependent; C, contralateral; dMRI, diffusion magnetic resonance imaging; fMRI, functional magnetic resonance imaging; I, ipsilateral; PD, Parkinson’s disease (adapted and reproduced with permission from Burciu et al. [119]).

The effects of antiparkinsonian medications on FWI metrics have also been elucidated. Chung et al. [118] found that there were no significant differences in FW and FA_T_ between the on- and off-medication phases, supporting the use of FWI metrics as disease progression biomarkers in PD. Furthermore, patients with PD on rasagiline, a monoamine oxidase type B inhibitor, exhibited lower FW in the posterior SN (Figure 8), together with a greater percentage of signal change in the posterior putamen during a force-producing task and a better performance on a coordination task, compared with patients who were not on rasagiline [119].

In patients with early-stage PD (Hoehn and Yahr stage 1–2), Andica et al. [42] evaluated the utility of FWI metrics in WM and GM using TBSS and GBSS analyses, respectively. The changes in FWI metrics were evident in more specific WM areas compared to the changes observed in DTI metrics. Notably, the changes in FWI metrics suggesting neurodegeneration (i.e., decreased FA_T_ and increased free water-corrected MD (MD_T_), free water-corrected AD (AD_T_), and free water-corrected RD (RD_T_)) and neuroinflammation (i.e., increased FW) were observed in the anterior and posterior WM areas, respectively. In line with the pathological trajectory of PD, these findings suggest that neuroinflammation precedes neurodegeneration in PD. At the same time, the patients with PD exhibited higher MD_T_, AD_T_, and FW in GM areas corresponding to Braak stage IV, without significant changes in DTI metrics.

**Figure 9:**
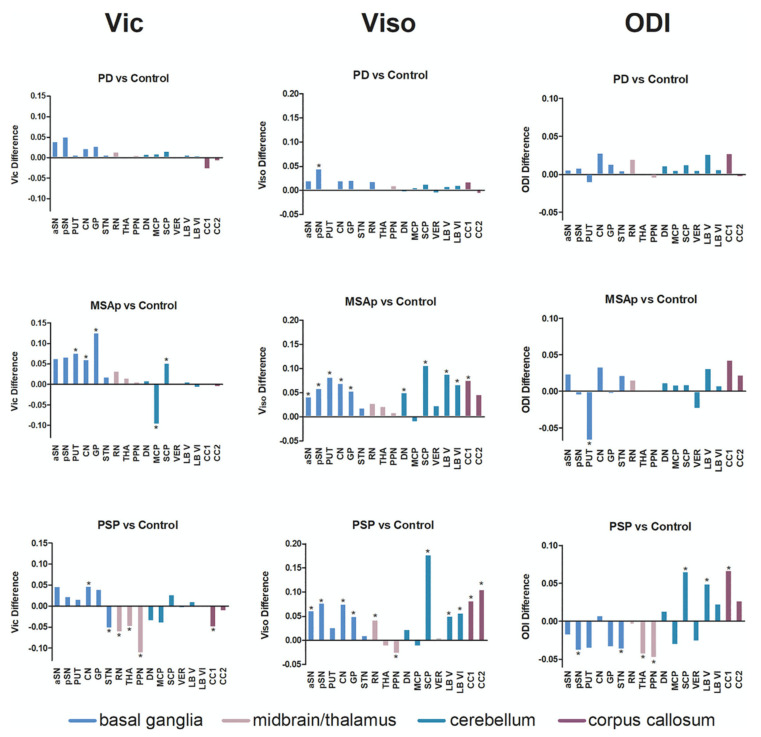
NODDI imaging in Parkinsonism. Between-group differences in patients with PD, MSAp, and PSP compared to controls in all regions of interest for each NODDI metric. * *p* < 0.05, false discovery rate-corrected. Abbreviations: aSN, anterior substantia nigra; CC1, prefrontal corpus callosum; CC2, premotor corpus callosum; CN, caudate nucleus; DN, dentate nucleus; GP, globus pallidus; LB V, cerebellar lobule V; LB VI, cerebellar lobule VI; MCP, middle cerebellar peduncle; ODI, orientation dispersion index; PPN, pedunculopontine nucleus; pSN, posterior substantia nigra; PUT, putamen; RN, red nucleus; SCP, superior cerebellar peduncle; STN, subthalamic nucleus; THA, thalamus; VER, cerebellar vermis; Vic, intracellular volume fraction; Viso, isotropic volume fraction (adapted and reproduced with permission from Mitchell et al. [120]).

FW can also be utilized to differentiate PD from diseases with atypical Parkinsonism diseases, such as progressive supranuclear palsy (PSP) and multiple system atrophy (MSA) [114,120]. Compared to controls, elevated FW is observed in the anterior and posterior SN in patients with PD, PSP, and MSA (Figure 9). However, only patients with PSP and MSA exhibit widespread increases in FW beyond SN, including the thalamus, basal ganglia, midbrain, and cerebellum. Furthermore, increased FA_T_ has been shown only in the basal ganglia of patients with PSP and MSA. A comparison of the FWI and NODDI metrics in terms of their ability in differentiating PD from atypical parkinsonism is elucidated in the next section (Section 3.2.4 NODDI in PD).

#### 3.2.4. NODDI in PD

The use of NODDI for the diagnosis and detection of disease progression in PD has been widely demonstrated [121]. Kamagata et al. [122] reported decreased ICVF and ODI in the contralateral SN pars compacta of patients with PD compared to controls. Indeed, patients with PD exhibit symptom unilaterality at disease onset, when the dominant side is strongly correlated with dopaminergic neuron loss in the contralateral hemisphere [123]. Furthermore, in a study by Kamagata et al., ROC analysis indicated that ICVF (area under the ROC curve (AUC), 0.92; sensitivity, 0.88; specificity, 0.83) in the contralateral SN pars compacta had a better diagnostic ability compared with the DTI measures [122]. The ICVF and ODI values in the SN pars compacta were also negatively correlated with the UPDRS-III score used for the severity of PD motor symptoms [122].

The loss of dopaminergic neurons in SN is considered to reduce the dopaminergic function of connections from the SN pars compacta to the corpus striatum, known as the nigrostriatal pathway, leading to the characteristic motor signs of PD [124]. Andica et al. [125] and Guo et al. [126] used NODDI to report decreased ICVF and ODI, respectively, in the nigrostriatal pathways of patients with PD compared to the controls. Interestingly, the changes in ICVF were only observed in the contralateral distal part of the nigrostriatal pathway, suggesting retrograde degeneration [125]. Furthermore, Guo et al. [126] reported decreased ODI, together with a trend of increasing FA in the contralateral amygdala-accumbens-pallidum pathway, which suggested hyperconnectivity related to pathological progression.

**Figure 10:**
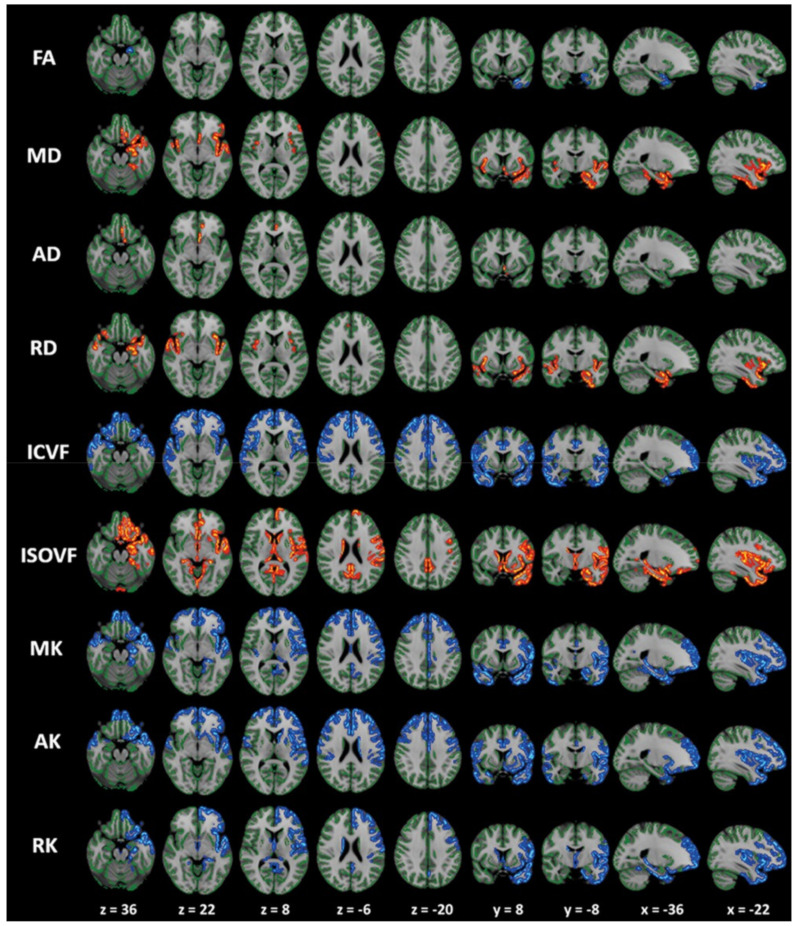
Areas with significant differences between patients with PD and controls based on GBSS analysis of the DTI, DKI, and NODDI metrics. GBSS analysis shows decreased FA, ICVF, MK, AK, and RK (blue-light blue voxels) and increased MD, AD, RD, and ISOVF (red-yellow voxels) in patients with PD compared with age-matched controls. All images are displayed according to neurological conventions on the Montreal Neurological Institute template. In patients with PD, the cortical GM in the limbic, paralimbic, frontal, and temporal areas demonstrated significantly decreased RK, MK, AK, and ICVF in comparison with the control group (GBSS analysis). Areas with significant changes in conventional DTI parameters (FA, AD, and RD) are visibly smaller than those with significant changes in RK, MK, AK, and ICVF. Results with significance (corrected *p* < 0.05) were thickened using the fill script implemented in FSL to improve visualization. Abbreviations: AD, axial diffusivity; AK, axial kurtosis; FA, fractional anisotropy; GBSS, gray matter-based spatial statistics; GM, gray matter; ICVF, intracellular volume fraction; ISOVF, isotropic volume fraction; MD, mean diffusivity; MK, mean kurtosis; OD, orientation dispersion index; PD, Parkinson’s disease; RD, radial diffusivity; RK, radial kurtosis (adapted and reproduced with permission from Kamagata et al. [28]).

Kamagata et al. [28] evaluated NODDI and DKI measures in the GM using ROI and GBSS analysis. The authors found that patients with PD changes in DKI (decreased MK, AK, and RK) and NODDI (decreased NDI and increased ISOVF) metrics in the limbic, paralimbic, frontal, and temporal cortices that corresponded to Braak stages IV and V, compared to controls (Figure 10); these results reflected neuronal loss due to the inhibition of GM neurite outgrowth and branching, as well as sparse neurite structure. Although the patients with PD displayed decreased FA and increased MD, AD, and RD, the changes in DTI metrics were more circumscribed compared with the broader abnormalities observed with the NODDI and DKI metrics. Thus, NODDI and DKI appear to have greater sensitivity than DTI for the detection of GM abnormalities in PD. Linear discriminant analysis also indicated that MK and ICVF maximized the predictive diagnostic accuracy in PD. Additionally, changes in NODDI and DKI metrics in the basal ganglia and the limbic, paralimbic, frontal, and temporal areas were correlated with the UPDRS-III score.

**Figure 11:**
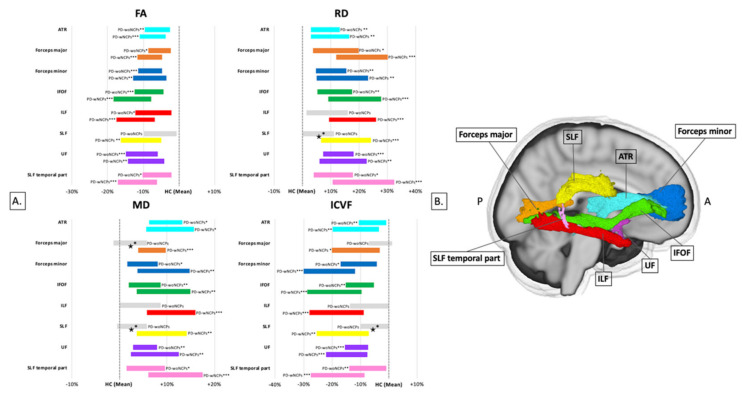
Significant tracts revealed by tract-of-interest analysis for comparison of diagnostic groups. Mean of each measure in the PD-woNCPs and PD-wNCPs groups, represented as percentage difference from healthy controls (**A**). Non-significant tracts are shown in gray, whereas significant tracts (* *p* < 0.05, ** *p* < 0.01, *** *p* < 0.001) are displayed in other colors. ^★^ Tracts with significant differences between the PD-woNCPs and PD-wNCPs groups. Tracts obtained using the ICBM-DTI-81 white matter tractography atlas (**B**). Abbreviations: ATR, anterior thalamic radiation; FA, fractional anisotropy; HC, healthy control; ICVF, intracellular volume fraction; IFOF, inferior fronto-occipital fasciculus; ILF, inferior longitudinal fasciculus; MD, mean diffusivity; PD-woNCPs, Parkinson’s disease without neurocognitive and psychiatric symptoms; PD-wNCPs, Parkinson’s disease with neurocognitive and psychiatric symptoms; RD, radial diffusivity; SLF, superior longitudinal fasciculus; UF, uncinate fasciculus (adapted and reproduced with permission from Andica et al. [127]).

PD is associated with a wide variety of non-motor symptoms. Patients with PD who experience neurocognitive and psychiatric disorders (i.e., cognitive impairment, hallucinations, depression, anxiety, apathy, and dopamine dysregulation syndrome) exhibit broader axonal loss (indexed by ICVF), predominantly in the posterior regions of the WM, compared to those without these symptoms (Figure 11) [127]. Using linked independent component analysis, Andica et al. [127] demonstrated that ICVF was the highest contributor to diagnosis (59% weight) when compared to the DTI metrics (FA, MD, AD, and RD), ODI, and WM volume.

Mitchell et al. [120] combined NODDI and FWI data collected from multiple study sites for the evaluation of PD and atypical Parkinsonism. In line with the results on FWI, higher ISOVF was detected in the posterior SN in PD and both the anterior and posterior SN in atypical Parkinsonism compared to controls. ICVF, ODI, and ISOVF were also altered in in the basal ganglia, midbrain, cerebellum, and corpus callosum in PSP and MSA compared to controls. FWI (AUC, 0.969) and NODDI (AUC, 0.945) were able to accurately discriminate PD from atypical Parkinsonism.

**Figure 12:**
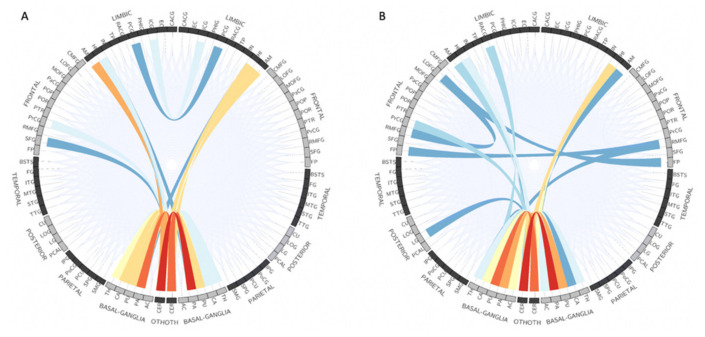
Top 30 neural connections determined by Grad-CAM analysis for differentiating patients with PD from healthy controls with ICVF-weighted (**A**) and AVF-weighted (**B**) connectome matrices. The more intensely focused connections are represented in a more reddish color. Abbreviations: AVF, axonal volume fraction; Grad-CAM, gradient-weighted class activation mapping; ICVF, intracellular volume fraction; PD, Parkinson’s disease (adapted and reproduced with permission from Yasaka et al. [128]).

Recently, Yasaka et al. [128] applied a convolutional neural network model to test the diagnostic performance of diffusion-weighted connectome matrices of DTI, DKI, and NODDI in differentiating patients with PD from controls. Overall, the DKI-weighted connectome performed better (AUC, 0.878–0.895) than the other matrices. However, using gradient-weighted class activation mapping (i.e., Grad-CAM), the ICVF-weighted connectome matrix was able to show the difference in connections between the basal ganglia and cerebellum in patients with PD compared to controls (Figure 12). In contrast, no specific trend was found in the areas of focus for neural connections with the RK-weighted connectome matrix.

### 3.3. Amyotrophic Lateral Sclerosis

Amyotrophic lateral sclerosis (ALS) is a neurodegenerative disease characterized by progressive muscular weakness due to the loss of motor neuron function involving both upper and lower motor neurons [129]. The pathogenic mechanisms in ALS are not well understood; however, a positive family history for ALS can be detected in 5–10% of patients with ALS and repeat expansion in the chromosome 9 open reading frame 72 gene (C9ORF72) is the most common cause of familial ALS [129]. The diagnosis of ALS is based on clinical symptoms, and there are currently no biomarkers approved for the diagnosis and prognosis of ALS. Therefore, the identification of noninvasive neuroimaging biomarkers is important. MRI, which has been extensively evaluated in patients with ALS for its utility, has provided important insights on morphological and structural changes in ALS. Specifically, recent developments in diffusion MRI have enabled the detection of microstructural abnormalities and several diffusion MRI techniques have been utilized for the assessment of diagnosis and prognosis in ALS.

#### 3.3.1. DTI in ALS

DTI is the most extensively researched diffusion MRI technique used to infer WM structural alterations within the brain and spinal cord of patients with ALS. Two voxel-based meta-analyses revealed that FA was consistently decreased in the corticospinal tract, posterior limb of internal capsule, and cingulate gyrus in patients with ALS [130,131]. FA reduction was also demonstrated in the corpus callosum; this change extended to primary motor cortices [131,132]. Additionally, the ALS Functional Rating Scale-Revised (ALSFRS-R) score, a popular metric used to evaluate the functional status of patients with ALS, was positively correlated with FA reduction in the left corona radiata [131]. Based on whole-brain-based spatial statistical analysis of patients with ALS harboring C9ORF72 mutations, decreased FA was observed mainly along the CST, with projections to frontal and hippocampal areas. In addition, tractwise analysis of differences in FA between patients with ALS harboring C9ORF72 mutations and controls revealed significant reductions in in the corticorubral and corticopontine tracts, the corticostriatal pathway, and the proximal perforant path [133]. These studies provide evidence that neuronal degeneration in ALS involves not only motor but also extramotor areas. Thus, DTI appears to be one of the most useful methods for unveiling microstructural changes in WM; however, these studies used a single-tensor diffusion model, which was a limiting factor in the characterization of heterogeneous microstructures in brain tissues and in the representation of important pathological features of ALS.

#### 3.3.2. DKI in ALS

**Figure 13:**
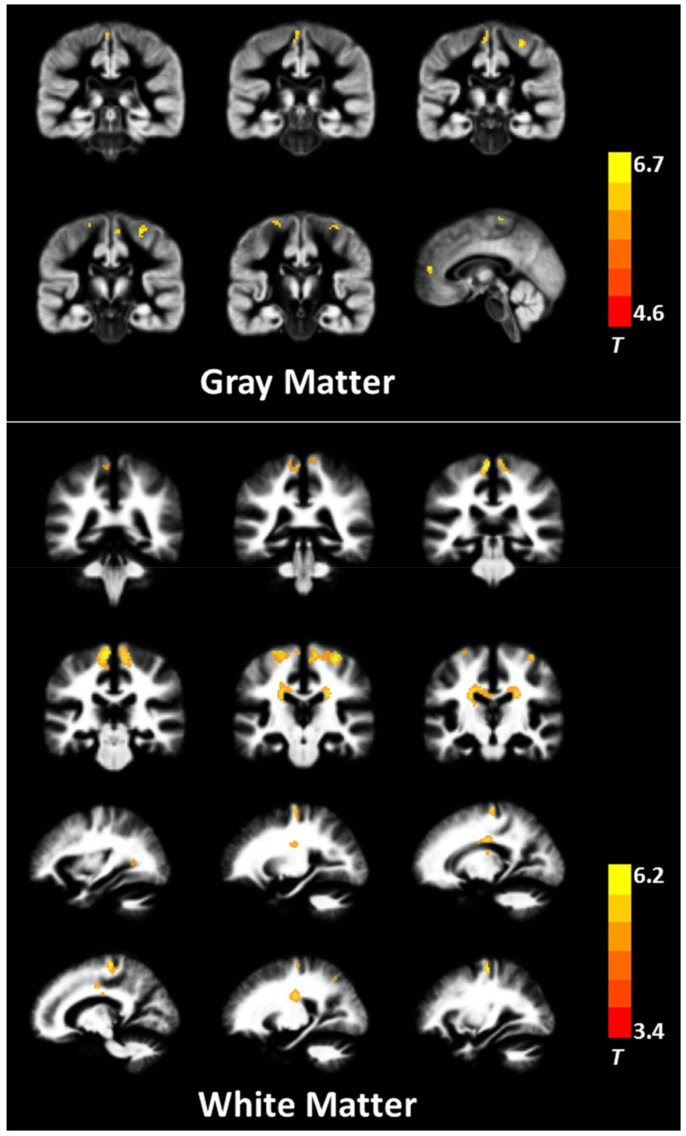
GM and WM regions with significantly decreased MK in ALS patients. The images displayed are overlaid on the averaged GM and WM maps derived from all subjects. Color bar represents T-values to indicate the difference in MK between patients with ALS and controls. Abbreviations: ALS, amyotrophic lateral sclerosis; GM, gray matter; MK, mean kurtosis; WM, white matter (adapted and reproduced with permission from Huang et al. [134]).

DKI allows the simultaneous microstructural characterization of both GM and WM, which is not possible with DTI because DKI is independent of the spatial direction of structures. Therefore, DKI may be useful in providing complementary information on the whole-brain microstructure in ALS. For example, patients with ALS exhibit decreased MK and RK in WM, including the bilateral precentral gyri, bilateral corona radiata, bilateral middle corpus callosum, left occipital lobe, and right superior parietal lobule compared to controls, based on voxel-based analysis. In GM regions, MK is decreased in the bilateral precentral gyri, bilateral paracentral lobules, and left anterior cingulate gyrus of patients with ALS (Figure 13). Additionally, several WM regions also exhibit decreased FA or increased MD/RD; however, the spatial extent is smaller than that observed using the decreased DKI metrics.

In one study, MK of the right precentral gyrus and RK of the left caudate body were positively correlated with the ALSFRS-R score, whereas MK and RK in the left precentral gyrus were negatively correlated with disease duration [134]. In another study, ROI analysis was used to demonstrate significant differences in the motor cortex between the ALS (contralateral to the symptomatic limb) and control (average of left and right) groups. MK, AK, and RK were significantly lower in the ALS group, whereas the conventional DTI measures were not significantly different between the two groups. Additionally, RK was positively related with the ALSFRS-R score [135]. Both studies demonstrated reductions in MK and RK in patients with ALS. A reduction in RK reflects myelin alterations in the brain and may therefore indicate myelin impairment in patients with ALS.

#### 3.3.3. FWI in ALS

To our knowledge, there is no previous published research to date that has investigated the utility of FWI in ALS.

#### 3.3.4. NODDI in ALS

**Figure 14:**
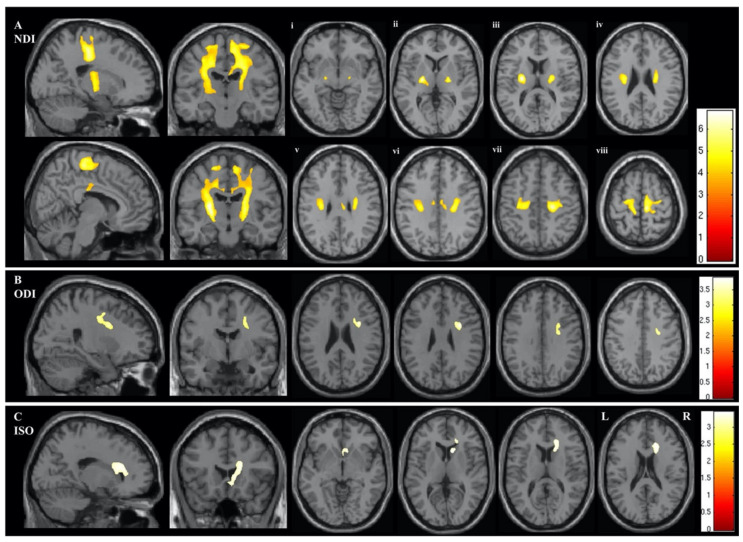
Areas showing significant differences in the whole-brain NODDI parameters of NDI (**A**), ODI (**B**), and ISO (**C**) between ALS and control groups. The results are shown using a statistical significance of *p* < 0.05 after family-wise error correction at the cluster level, with the clusters created using a *p*-value of <0.001. Panels **Ai**–**viii** show the areas with significant differences in NDI on axial sections from the posterior limb of the internal capsule (**vi**) extending rostrally up into the subcortical WM of the precentral gyrus (**viii**). Abbreviations: ALS, amyotrophic lateral sclerosis; ISO, isotropic component; NDI, neurite density index; NODDI, neurite orientation dispersion density imaging; ODI, orientation dispersion index; WM, white matter (adapted and reproduced with permission from Broad et al. [137]).

Despite its recent introduction to the evaluation of patients with ALS, NODDI may be more sensitive than DTI. Indeed, in a whole-brain voxelwise analysis using NODDI, the ALS group demonstrated significantly decreased NDI in extensive regions of the corticospinal tract, corpus callosum, and right precentral gyrus, decreased ODI in right anterior internal capsule and right precentral gyrus, and increased ISO within the right lateral ventricle (Figure 14) [136]. Moreover, significantly decreased NDI was found in the corona radiata and subcortical WM of the right hemisphere in patients with both bulbar and limb involvement compared to those with limb-only involvement. Decreased ODI in the precentral gyri, precuneus, and dorsolateral prefrontal cortex was correlated with disease duration. In a study using whole-brain analysis by means of DTI, the ALS group exhibited decreased FA within the corticospinal tract, although the affected areas were more limited compared with those determined by NDI [137].

**Figure 15:**
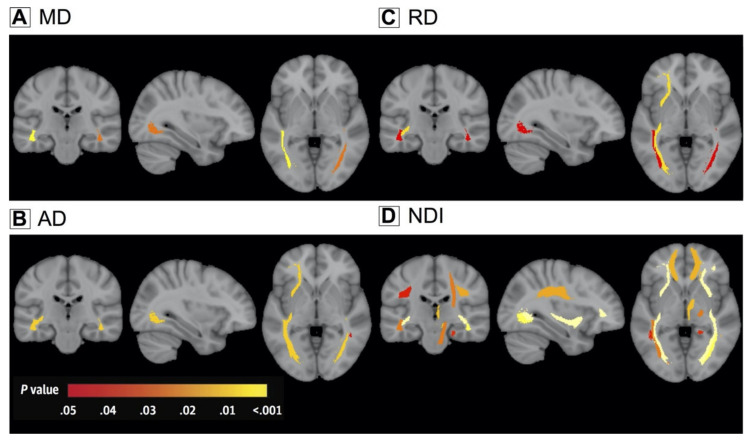
White matter alterations in C9ORF72 mutation carriers. Color-coded representation of *p*-values corresponding to the association of C9ORF72 mutation with white matter integrity after correction for multiple comparisons. MD (**A**), AD (**B**), RD (**C**), and NDI (**D**). Abbreviations: AD, axial diffusivity; MD, mean diffusivity; NDI, neurite density index; RD, radial diffusivity (adapted and reproduced with permission from Wen et al. [138]).

A study in which researchers performed ROI analysis of NODDI and DTI in premanifest C9ORF72 mutation carriers revealed that, compared with the noncarriers, C9ORF72 mutation carriers demonstrated ND-WM abnormalities in 10 tracts, including the corticospinal tract, based on NDI, and in only five tracts based on the DTI metrics (increased AD, RD, and MD rather than decreased FA) (Figure 15). In two tracts, the effect size was significantly higher for NDI compared with the DTI metrics. ISOVF was increased in 13 regions, whereas 11 regions displayed volumetric atrophy in the C9ORF72 mutation carriers. These results corroborate the suggestion that lower FA (or increased diffusivity) in the CST and corpus callosum is a result of axonal fiber loss rather than an increase in complexity or dispersion within the tracts [138].

Furthermore, a study which combined NODDI with the histopathological assessment of the manually segmented WM anterolateral region in the spinal cord of a rodent model of ALS (G93A-SOD1 mice) [136] showed decreased NDI and increased ODI both in presymptomatic (early stage) and symptomatic (later stage) mice compared with the control mice. In the same study, a statistically significant histological reduction in total axonal area was observed in the early stage. Additionally, a significant expansion in the extra-axonal compartment was observed in the early disease stage; a further decrease in axonal areas and an increase in the extra-axonal compartment were observed in later disease stages. Decreased NDI reflected a reduction in axonal area and myelin content, based on histopathological evaluation. Overall, these studies suggest that NODDI can detect WM abnormalities caused by axonal degeneration in ALS.

## 4. Conclusion and Future Directions

Advanced diffusion MRI techniques, including FWI, DKI, and NODDI, which provide new findings on brain microstructures, have contributed to the elucidation of neurodegenerative diseases. Nonetheless, these advanced techniques have yet to be introduced in clinical settings, as in the routine use of DWI to assess acute infarction, due to the lack of clinical evidence on their utility. Moreover, unlike the measurement of hippocampal volume, a biomarker for AlzD that is utilized to decrease the necessary sample size and cost of clinical trials in the detection of neurodegenerative changes [139], the evidence is insufficient regarding the utility of advanced diffusion MRI-based biomarkers in neurodegenerative diseases. The high associated cost also hinders the implementation of clinical trials. Thus, the low-cost efficacy of advanced diffusion MRI techniques hampers the accumulation of clinical evidence.

Several limitations must be addressed to achieve the clinical utilization of advanced diffusion MRI techniques for the diagnosis of neurodegenerative diseases. First, the relationship between pathological changes in neurodegenerative diseases and advanced diffusion MRI metrics remains unclear. DKI, FWI, and NODDI only model and predict brain microstructures using diffusion MRI, and the extent to which these models can reflect and explain specific neurodegenerative diseases is unknown. Therefore, further studies on neurodegenerative diseases in postmortem human tissues or animal models are needed to clarify the relationship between pathological findings and the advanced diffusion MRI metrics of FWI, DKI, and NODDI. Second, the reproducibility and reliability of the results of studies using advanced diffusion MRI techniques are quite low because of the low statistical power caused by small sample sizes. Therefore, the utility of FWI, DKI, and NODDI as biomarkers for neurodegenerative diseases should be established based on strong evidence from multi-site studies with larger sample sizes to improve their statistical power. Although several large-scale multi-site studies are underway, MRI scanners and acquisition parameters are highly diverse and depend on the specific imaging site [140]. These differences across study sites might lead to low reproducibility and reliability in advanced diffusion MRI studies. For example, Andica et al. [141] evaluated the scan-rescan and inter-vendor reproducibility of DTI and NODDI using two 3-T MRI scanners from two different vendors. The scan-rescan coefficient of variation of NODDI metrics using both scanners was comparable with that of DTI metrics (0.2–3.8% and 0.1–4.1%, respectively). However, the inter-vendor CoV was higher than the scan-rescan CoV for NODDI metrics (2.3–14%). Moreover, the inter-sequence variability of DTI metrics using three different sequences showed that the CoVs for FA and MD were 5.45–7.34% and 1.72–5.56%, respectively [142]. Furthermore, Kamagata et al. [143] evaluated the inter-site reliability of DTI metrics using identical 3-T MRI scanners at two different sites and acquisition parameters. The authors reported that the CoV of DTI ranged from 0.6 to 5.6%. Thus, variations in diffusion MRI metrics caused by site differences, including MRI scanners and acquisition parameters, may reduce their statistical power, leading to low reproducibility and reliability in multi-site studies using advanced diffusion MRI techniques [140]. In particular, changes in diffusion MRI metrics in neurocognitive and psychiatric disorders are subtle (approximately 5–6%) compared to healthy controls and on the same order as that of site difference; therefore, it is difficult for a multi-site study to detect pathological changes in patients with neurocognitive and psychiatric disorders [144,145,146]. Therefore, it is necessary to reduce inter-site variability in diffusion MRI metrics by standardizing MRI methods including MRI scanners and acquisition parameters and harmonizing multi-site diffusion MRI data. For example, several harmonization methods for diffusion MRI have been proposed, such as a combined association test called ComBat [147], linear regression based on rotation invariant spherical harmonics [148], and the deep learning approach [149], to reduce the variability between MRI scanners and protocols. ComBat uses the regression of covariates for the data harmonization of diffusion MRI metrics with an empirical Bayes inference. Linear rotation invariant spherical harmonics uses features for diffusion MRI signal harmonization and for mapping of the diffusion MRI data from a target site to a reference site. The deep learning harmonization method optimizes neural network parameters using diffusion MRI signals obtained in target and reference sites in the learning stage and then harmonizes diffusion MRI data using the trained neural network. These harmonization techniques not only reduce unwanted variations in DTI metrics caused by site differences but also preserve biological variability caused by age and sex.

Another limitation is the unclear relationship between the pathological changes induced by neurodegenerative diseases and advanced diffusion MRI metrics. DKI, FWI, and NODDI can only model and predict brain microstructures using diffusion MRI, and the extent to which these models can reflect and explain specific neurodegenerative diseases is unknown. Therefore, further studies of neurodegenerative diseases in postmortem human tissues or animal models are needed in order to clarify the relationship between pathological findings and the advanced diffusion MRI metrics of DKI, FWI, and NODDI. Resolving these limitations should lead to the clinical utilization of advanced diffusion MRI techniques as biomarkers for the diagnosis of neurodegenerative diseases in clinical settings.

Recently, frameworks that combine diffusion tensor MRI and relaxometry have been introduced to improve their specificity for the quantification of myelin and axonal attributes irrespective of the complexity of fiber organization within the voxel, even in the presence of crossing fibers [150,151]. A growing body of evidence also suggests the role of excess iron deposition in the pathology of neurodegenerative diseases. Quantitative susceptibility mapping is a promising imaging technique for the comprehensive investigation of iron distribution in the brain [152]. The use of the diffusion tensor-relaxometry framework or combining advanced diffusion MRI techniques and quantitative susceptibility mapping might provide a more comprehensive picture of neurodegenerative diseases by analyzing different biological tissue properties.

## Figures and Tables

**Table 1:** Summary of the characteristics of diffusion indices.

Model	Indices	Summary of Models	Implication	Unique Limitation
DTI	FA	Signal representation that estimates the anisotropy and directionality of water diffusion by assuming a single ellipse in a voxel	Overall directionality of water diffusion within the brain tissue	Partial volume effect associated with CSF contaminationNonspecificity due to the lack of inclusion of biophysical assumptions
	MD		Magnitude of isotropic diffusion within the brain tissue	
	AD		Magnitude of isotropic diffusion along the main axis	
	RD		Magnitude of isotropic diffusion along the transverse direction	
DKI	MK	Signal representation that quantifies the degree of non-Gaussian diffusion associated with complex brain microstructures	Degree of diffusion restriction or brain tissue complexity	Long scanning time to acquire at least two b-values Nonspecificity due to the lack of inclusion of biophysical assumptions
	AK		Degree of diffusion restriction or brain tissue complexity along the main axis of maximal diffusion	
	RK		Degree of diffusion restriction or brain tissue complexity along the transverse direction	
FWI	FW	Two-compartment model that quantifies extracellular free water to eliminate indices contaminated by CSF from DTI	Free water within extracellular space	High dependence of single-shell FWI model fitting on regularization constraints
	Free water-corrected DTI		DTI parameters, eliminating the influence of extracellular free water	
NODDI	ICVF	Three-compartment model that represents neurite density, isotropic free water, and dispersion of neurites (e.g., axons, dendrites, and others)	Neurite density within a voxel based on the intracellular compartment	Long scanning time to acquire at least two b-values for highly accurate parameters
	ISOVF		Volume fraction of isotropic diffusion based on the extracellular compartment	
	ODI		Orientation dispersion of neurites based on the intracellular compartment	

Abbreviations: AD, axial diffusivity; AK, axial kurtosis; DKI, diffusion kurtosis imaging; DTI, diffusion tensor imaging; FA, fractional anisotropy; FW, free-water volume fraction; FWI, free-water imaging; ICVF, intracellular volume fraction; ISOVF, isotropic volume fraction; MD, mean diffusivity; MK, mean kurtosis; NODDI, ODI, orientation dispersion index; RD, radial diffusivity; RK, radial kurtosis.

## Data Availability

Not applicable.

## Abbreviations

Aβ	Amyloid-β
AD	Axial diffusivity
AD_T_	Free water-corrected axial diffusivity
AK	Axial kurtosis
ALS	Amyotrophic lateral sclerosis
ALSFRS-R	Amyotrophic lateral sclerosis functional rating scale-revised
AlzD	Alzheimer’s disease
APOE	Apolipoprotein E
AUC	Area under the receiver-operating characteristics curve
C9ORF72	Chromosome 9 open reading frame 72 gene
COV	Coefficient of variation
CSF	Cerebrospinal fluid
DKI	Diffusion kurtosis imaging
DTI	Diffusion tensor imaging
FA	Fractional anisotropy
FA_T_	Free water-corrected fractional anisotropy
FWI	Free-water imaging
GM	Gray matter
GBSS	Gray matter-based spatial statistics
ICVF	Intracellular volume fraction
ISOVF	Isotropic volume fraction
MCI	Mild cognitive impairment
MD	Mean diffusivity
MD_T_	Free water-corrected mean diffusivity
MK	Mean kurtosis
MMSE	Mini-mental state examination
MRI	Magnetic resonance imaging
MSA	Multiple system atrophy
NODDI	Neurite orientation dispersion and density imaging
ODI	Orientation dispersion index
PD	Parkinson’s disease
PET	Positron emission tomography
PSP	Progressive supranuclear palsy
RD	Radial diffusivity
RD_T_	Free water-corrected radial diffusivity
RK	Radial kurtosis
ROC	Receiver-operating characteristics
ROI	Region of interest
SN	Substantia nigra
TBSS	Tract-based spatial statistics
WM	White matter
UPDRS	Unified Parkinson’s disease rating scale
